# Multiple concomitant mechanisms contribute to low platelet count in patients with immune thrombocytopenia

**DOI:** 10.1038/s41598-018-38086-1

**Published:** 2019-02-18

**Authors:** Matías Grodzielski, Nora P. Goette, Ana C. Glembotsky, M. Constanza Baroni Pietto, Santiago P. Méndez-Huergo, Marta S. Pierdominici, Verónica S. Montero, Gabriel A. Rabinovich, Felisa C. Molinas, Paula G. Heller, Paola R. Lev, Rosana F. Marta

**Affiliations:** 1 University of Buenos Aires, Institute of Medical Research A Lanari, Buenos Aires, Argentina; 2 Department of Experimental Hematology, Institute of Medical Research (IDIM), National Scientific and Technical Research Council (CONICET), University of Buenos Aires, Buenos Aires, Argentina; 3 Laboratory of Immunopathology, Institute of Biology and Experimental Medicine (IBYME), National Scientific Council of Scientific and Technical Research (CONICET), C1428 Buenos Aires, Argentina; 4 Department of Hematology, Ramos Mejía Hospital, Buenos Aires, Argentina; 5 Department of Biochemistry, Center of Medical Education and Clinical Research “Norberto Quirno” (CEMIC), Buenos Aires, Argentina; 6 Department of Biological Chemistry, School of Exact and Natural Science, University of Buenos Aires, C1428 Buenos Aires, Argentina

## Abstract

Mechanisms leading to low platelet count in immune thrombocytopenia (ITP) involves both decreased production and increased destruction of platelet. However, the contribution of these pathologic mechanisms to clinical outcome of individual patients is uncertain. Here we evaluated different pathogenic mechanisms including *in vitro* megakaryopoiesis, platelet/megakaryocyte (MK) desialylation and MK apoptosis, and compared these effects with thrombopoyesis and platelet apoptosis in the same cohort of ITP patients. Normal umbilical cord blood-CD34+ cells, mature MK derived cells or platelets were incubated with plasma from ITP patients. Despite inhibition of thrombopoiesis previously observed, megakaryopoiesis was normal or even increased. Plasma from ITP patients affected the sialylation pattern of control platelets and this effect occurred concomitantly with apoptosis in 35% ITP samples. However, none of these abnormalities were observed in control MKs incubated with ITP plasma. Addition of mononuclear cells as immune effectors did not lead to phosphatidylserine exposure in MK, ruling out an antibody-mediated cytotoxic effect. These results suggest that both desialylation and apoptosis may be relevant mechanisms leading to platelet destruction although, they do not interfere with MK function. Analysis of these thrombocytopenic factors in individual patients showed no specific distribution pattern. However, the presence of circulating antiplatelet autoantibodies was associated with higher incidence of abnormalities. In conclusion, the causes of thrombocytopenia are multifactorial and may occur together, providing a rational basis for the use of combination therapies targeting concomitant ITP mechanisms in patients with refractory disease.

## Introduction

Primary immune thrombocytopenia (ITP) is defined as a megakaryocytic/platelet-specific autoimmune disorder characterized by isolated platelet count < 100 × 10^9^/L with or without bleeding manifestations, in the absence of other disorders that may be associated with thrombocytopenia^1^.

Mechanisms leading to low platelet count in ITP are multifactorial involving both, increased peripheral platelet destruction and decreased platelet production. Platelet destruction was classically described in the spleen through Fc–FcγR interactions in macrophages. In addition, Li and colleagues^2^ reported clearance of desialylated platelets in the liver via the hepatocyte Ashwell–Morell receptors. Recently, evidence from our group^3^ and others^4–6^ demonstrated increased platelet apoptosis in some ITP patients. These findings implied antibody-dependent cell cytotoxicity as a contributing mechanism leading to platelet apoptosis and peripheral clearance of ITP platelets. Concerning impaired platelet production, insufficient megakaryopoiesis was observed by McMillan^7^ and Chang^8^ in independent studies. In addition, we recently described impaired proplatelet formation (PPF)^9^ as a new mechanism leading to thrombocytopenia in these patients, results that were further confirmed^10^. Main factors responsible for ITP abnormalities are autoantibodies targeting MKs and platelet-specific glycoproteins as well as cytotoxic T cells directly acting on platelets.

The contribution of each one of these pathogenic mechanisms to thrombocytopenia in individual patients is unknown, as these pathways, to the best of our knowledge, have not been studied concurrently in the same ITP population. In addition, the effect of plasma from ITP samples on MK desialylation is still unexplored.

Classical treatment in ITP is aimed at modulating the abnormal immune response by the use of corticosteroids, intravenous immunoglobulin (IVIG), IV anti-D and rituximab, among others. More recently, therapy aimed at increasing platelet production was incorporated and recommended as second and third-line treatment. These drugs include eltrombopag, an oral, synthetic non-peptide agonist that binds to the trans-membrane domain of the thombopoietin receptor^11^ and romiplostim, a peptibody that interacts with the extracellular domain of the thrombopoietin receptor^12^. Currently, therapeutic decisions are made on the basis of clinical features or patient preference rather than the specific pathological mechanisms involved in individual patients.

In the present study we investigated different immunopathogenic mechanisms operating in ITP patients that contribute to low platelet count, including those involving decreased platelet production (inhibition of megakaryopoiesis and thrombopoiesis) and increased platelet clearance (platelet apoptosis and desialylation) with the ultimate goal of providing a rational guide for implementation of tailored therapies to individual patients.

## Results

### Megakaryopoiesis is not impaired by ITP plasma

**Figure 1:**
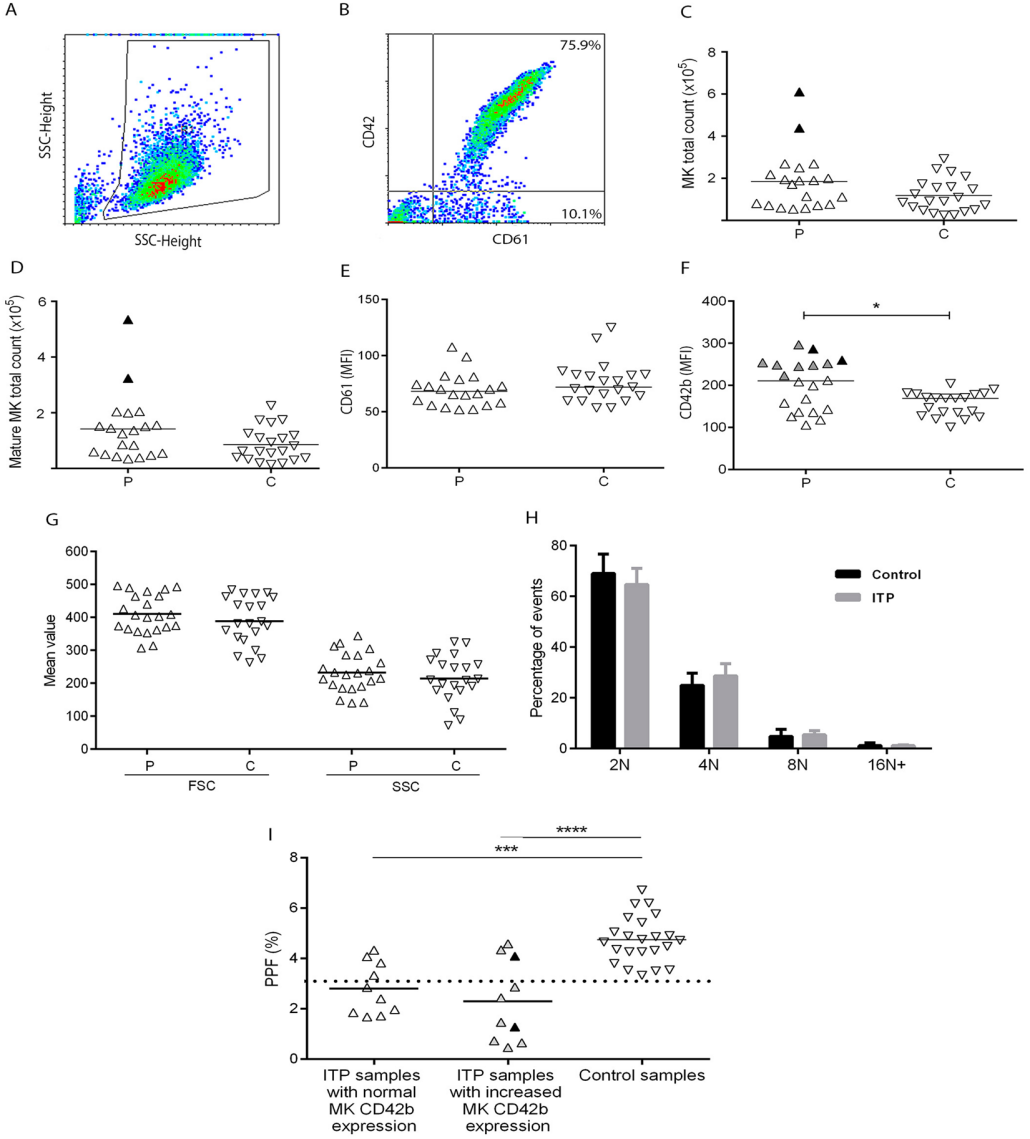
Megakaryocyte (MK) differentiation/maturation and proplatelet formation (PPF) in the presence of ITP plasma. CD34+ hematopoietic progenitors purified from normal cord blood were cultured in medium supplemented with thrombopoietin, interleukin 6 and 10% of either ITP (P) (n = 20) or control (C) (n = 23) recalcified plasma. After 12 days, cells were harvested, counted, labelled with FITC-CD61 and PE-CD42b and analysed by flow cytometry. (**A**) Cells were selected by forward scatter (FSC) versus side scatter (SSC) dot plot. (**B**) This gate was analysed for FITC-CD61 and PE-CD42b to determine MK commitment and maturation, respectively. (**C**) MK total count (CD61+ cells); Black triangles represent samples that induced increased MK cell count. (**D**) Mature MK total count (CD61+/CD42b+ cells). (**E**) and (**F**) mean fluorescence intensity (MFI) of CD61 and CD42b, respectively (*P < 0.05, Mann-Whitney test); Grey triangles represent samples that induced increased MK CD42b expression. (**G**) MK size and complexity were evaluated by FSC and SSC, respectively. (**H**) MK ploidy was assessed in cells incubated either with patient (ITP) or control plasma using propidium iodide. (**I**) Analysis of the effect of ITP plasma samples on PPF according to their ability to influence MK maturation. Percentage of proplatelet forming-MKs was measured by inverted microscopy (Axiovert 25; Carl Zeiss GmbH, G€ottingen, Germany) after 48 h-incubation of cord-blood derived mature (day 13) MKs with ITP or control plasma, as described^9^. Data are grouped according to the ability of ITP plasma to induce normal or increased MK maturation (***P < 0.001; ****P < 0.0001, one-way ANOVA followed by Tukey’s multiple comparisons test). Triangles represent mean value of at least two separate experiments using different cord blood samples. Horizontal lines and dotted line represent median or mean values and upper reference value, respectively.

In order to investigate the effect of plasma from our cohort of ITP patients on megakaryopoiesis, CD34+ progenitors isolated from normal cord-blood mononuclear cells (MNC) were incubated with 10% ITP or control recalcified plasma and analysed after 12 days. Total MK count (CD61+ cells) as well as mature MK count (CD61+/CD42+ cells) were normal in the presence of ITP plasma (Fig. 1A–D), although plasma from two ITP patients induced an increase in both measurements. While CD61 mean fluorescence intensity (MFI) was normal (Fig. 1E), CD42b MFI was higher in cells incubated with ITP plasma (Fig. 1F). In particular, nine samples induced an increase in CD42b MFI: one plasma corresponding to an ITP patient bearing autoantibodies targeting GPIbIX, three samples from ITP patients with autoantibodies directed against GPIIbIIIa and five samples negative for autoantibodies.

These assays were also carried out using CD34+ hematopoietic progenitors obtained from peripheral blood of mobilized normal subjects. Using this alternative source of control MKs, similar results were obtained (Supplementary Table S1).

Cell size and complexity, evaluated by mean forward scatter (FSC) and side scatter (SSC), and MK ploidy were similar in cells incubated with ITP and control plasma (Fig. 1G,H). Given that control MK CD42b expression was increased in the presence of ITP plasma, platelets from ITP patients were also tested for this specific marker. However, CD42b expression was not altered in platelets from our ITP cohort (data not shown).

### Relationship between the effect of ITP plasma on megakaryopoiesis and thrombopoiesis

We previously reported in an ITP cohort that patient’s plasma inhibits proplatelet formation (PPF) from normal cord blood-derived MKs and that this effect was reproduced using purified IgG^9^. In order to establish the relationship between the effect of ITP plasma on MK production and PPF, we compared the results on megakaryopoiesis obtained in the current study with our own reported data on thrombopoiesis using the same plasma samples. In particular, 13 out of 20 (65%) ITP samples inhibited thrombopoiesis, as evaluated by the percentage of MKs bearing proplatelets (unpaired *t* test, patients vs controls, p < 0.0001). Of note, while inhibition of thrombopoiesis was a frequent finding, these ITP samples did not impair megakaryopoiesis. Moreover, increased MK CD42b expression and decreased PPF were concomitantly observed in the presence of seven ITP samples: one of these also showed high total MK cell count (Fig. 1I). Thus, plasma-induced inhibition of platelet production in ITP patients involves a mechanism associated with platelet formation rather than MK development and maturation.

### Increased desialylation of control platelets in the presence of ITP plasma: relationship with platelet apoptosis

**Figure 2:**
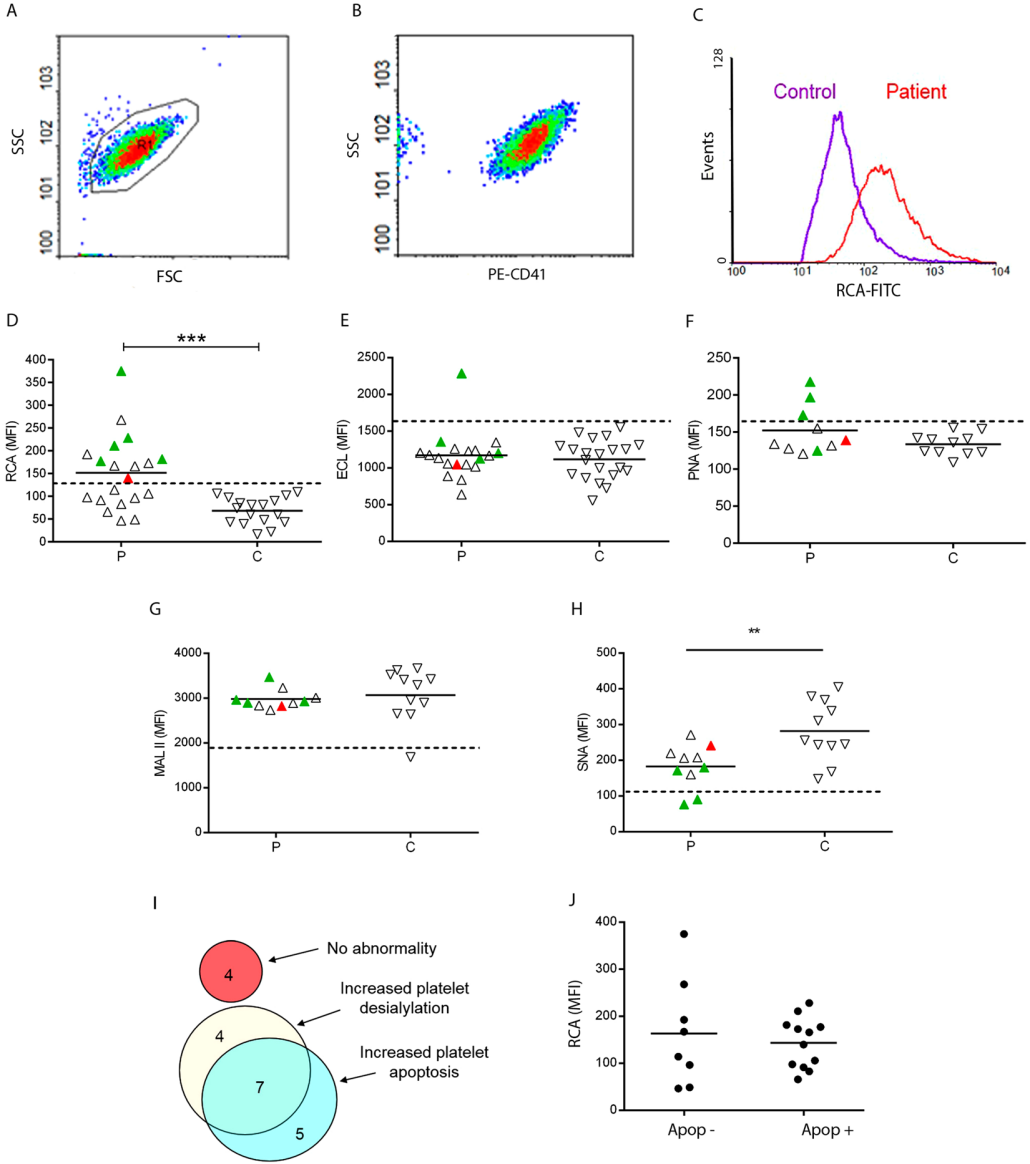
Platelet desialylation and apoptosis in ITP. To evaluate the pattern of sialylation in the presence of ITP plasma, control washed platelets were incubated with ITP (P) or control (**C**) recalcified plasma during 1 hour. After washing, isolated platelets were incubated with either FITC-RCA-I (P, n = 20; C, n = 17) or biotin-ECL (P, n = 19; C, n = 21), PNA, MAL II or SNA (P, n = 10; C, n = 11) followed by incubation with streptavidin-APC. Cells were acquired in a flow cytometer. (**A**) Cells were selected by FSC vs SSC. (**B**) This gate was analysed either for PE-CD41 or FITC-CD42a to identify the platelet population. Mean Florescence Intensity (MFI) of FITC-RCA from the PE-CD41 positive cells or APC-lectins from the FITC-CD42a positive cells were registered. (**C**) A representative example of RCA-binding to platelets incubated with an ITP plasma and a control plasma is shown. (**D**–**H**) Green triangles represent samples from ITP patients with autoantibodies against GPIIbIIIa; red triangles represent ITP sample with autoantibodies against GPIbIX. Horizontal lines represent mean values and dotted lines represent either upper (for RCA, ECL and PNA) or lower (for MALII and SNA) reference value. Samples were tested at least twice using different control platelet donors (**P < 0.001, Mann-Whitney test). (**I**) Venn diagram showing the number of patients/samples displaying increased platelet apoptosis and/or increased platelet desialylation. (**J**) MFI of FITC-RCA in ITP patients according to the presence (Apo+) or absence (Apo−) of platelet apoptosis.

To evaluate whether ITP plasma samples induce neuraminidase activity leading to loss of sialic acid from the platelet surface, control platelets were incubated with ITP plasma and their sialylation pattern was evaluated. Fifty five percent of ITP plasma samples induced increased binding of fluorescein-conjugated *Ricinus communis* agglutinin I (RCA-I) lectin to normal platelets. This lectin specifically binds to exposed galactose (Gal) residues (Galβ1-4GlcNAc) as a consequence of glycoprotein desialylation, (Fig. 2A–D). Interestingly, all ITP samples positive for autoantibodies induced an increase in RCA-I binding to normal platelets. Additionally, changes in platelet sialylation were further characterized using other lectins including *Erythrina cristagalli* lectin (ECL) which binds to galactosyl (β-1,4) N-acetylglucosamine (Galβ4GlcNAc) and peanut agglutinin (PNA) which recognizes galactosyl (β-1,3) N-acetylgalactosamine (Galβ3GalNAc; asialo core-1 O-glycans). Although there was not statistical difference between results obtained in the presence of ITP and control plasma, some samples induced an increase in lectin binding (Fig. 2E,F). Of interest, all these samples were from patients bearing anti-GPIIbIIIa autoantibodies.

In addition, we assessed the presence of α2,6-linked sialic acid attached to terminal galactose (Neu5Acα6Gal/GalNAc) by incubation with the *Sambucus nigra* agglutinin (SNA) and α2,3-linked sialic acid (Neu5Acα3Galβ4GalNAc) using *Maackia amurensis* lectin II (MAL II) as probes. While MAL II binding was not altered, SNA recognition decreased substantially when platelets were exposed to ITP plasma, demonstrating the involvement of α-2,6-linked sialic acid in this process (Fig. 2G,H).

In a previous study, we showed increased platelet apoptosis in an ITP cohort that also included patients evaluated in the present study^3^. Platelet apoptosis was considered to be increased in individual patients when at least two of three apoptosis parameters, including phosphatidylserine (PS) exposure, disruption of mitochondrial membrane potential and active caspase 3, were higher than the normal range. Twelve from the 20 ITP patients included in this study (60%) had increased platelet apoptosis^3^. Now, we compared our previous results on platelet apoptosis with those of desialylation evaluated by RCA binding, obtained in the present study. Of note, seven ITP samples (35%) induced both platelet apoptosis and desialylation, while others induced either apoptosis or desialylation (Fig. 2I). From the 7 ITP samples showing increased apoptosis and desialylation, 5 had detectable autoantibodies. On the contrary, four ITP samples behaved normal. The degree of platelet desialylation (as evaluated by MFI of RCA binding), did not differ between platelets incubated with ITP plasma from patients with increased apoptosis and those with normal apoptosis parameters (Fig. 2J).

Platelet activation in this ITP cohort was also assessed in our previous study^3^ using PAC-1 and CD62P binding to evaluate GPIIbIIIa activation and P-selectin externalization, respectively. Results obtained in patients were similar to normal controls suggesting that platelet desialylation would not be associated to activation in our ITP population.

### Desialylation and apoptosis of control MKs incubated with ITP samples

**Figure 3:**
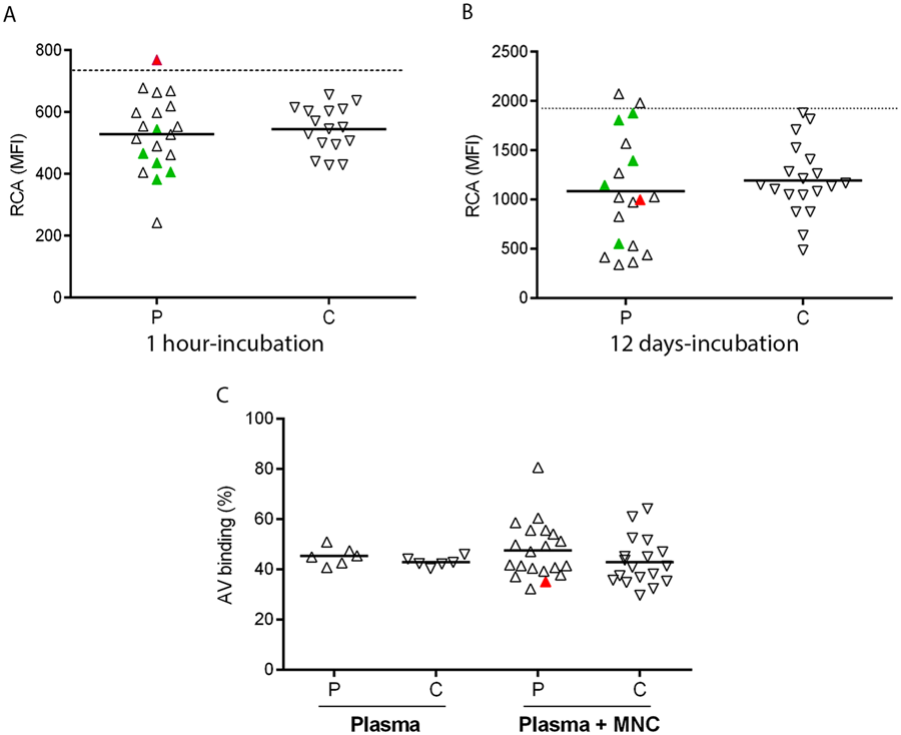
Desialylation and apoptosis of control megakaryocytes (MKs) induced by ITP plasma. (**A**) Control mature MK were obtained from normal CD34+ plasma-free cell culture with the addition of 10 ng/ml thrombopoietin and interleukin 6 for 12 days. Then, 10% ITP (P, n = 20) or control plasma (C, n = 16) were added and further incubated during 1 h. After that, MK desialylation was evaluated by FITC-RCA binding. (**B**) Normal CD34+ progenitors were culture in the presence of 10 µg/ml thrombopoietin and interleukin 6 and 10% ITP (P, n = 19) or control (C, n = 19) for 12 days. After that, MK desialylation was assessed as described in A). Mean Florescence Intensity (MFI) from the PE-CD41 positive cells is shown. Horizontal lines and dotted line represent median values and upper reference value, respectively. (**C**) For apoptosis studies, normal mature MK were incubated for 24 h with ITP (n = 6) or control (n = 6) plasma (Plasma) or with autologous mononuclear cells (MNC) in the presence of either ITP (n = 20) or control plasma (n = 18) (Plasma + MNC) and stained with FITC-annexin V. Triangles represent mean value of at least two separate experiments using different cord blood samples. In both graphs, red and green triangles represent the ITP sample with autoantibodies targeting GPIbIX and GPIIbIIIa, respectively.

To gain further insight into possible abnormalities generated by ITP autoantibodies in MKs, we extended the study of desialylation and apoptosis to control MKs incubated with recalcified ITP plasma. First, mature MKs were obtained from normal CD34+ plasma-free cell culture for 12 days. Then, 10% ITP or control plasma were added to the culture media and incubated for 1 h. MK desialylation assessed by RCA binding in these conditions was not altered in the presence of 95% of ITP samples (Fig. 3A). Interestingly, the only ITP sample that induced loss of sialic acid was the one bearing autoantibodies targeting GPIbIX (red triangle in Fig. 3A). Second, in order to obtain mature MKs developed in the presence of ITP samples, normal CD34+ progenitors were cultured in the presence of 10% ITP or control plasma during 12 days. RCA binding to these cells was not increased further (Fig. 3B).

We previously showed that ITP plasma does not trigger caspase 3 activation in control mature MKs^9^. To evaluate another parameter of cell apoptosis, we now tested annexin-V binding to MKs incubated with a restricted group of ITP samples (n = 6). Levels of PS exposure were not found to be altered (Fig. 3C). To evaluate whether ITP autoantibodies may induce antibody-dependent cell cytotoxicity (ADCC) leading to MK apoptosis, PS exposure was assessed after co-incubation of MKs with MNC in the presence of ITP or control plasma. To avoid HLA mismatch, CD34+ cells used for MK culture and MNC were obtained from the same donor sample. Under these conditions, PS exposure was increased in the presence of only one of 20 ITP samples, suggesting that autoantibodies do not alter mature MK survival by themselves or by the indirect action of immune effector cells (Fig. 3C). The ITP sample containing autoantibodies that target GPIbIX did not induce significant alterations under these conditions (red triangle in Fig. 3C).

### Coexistence of factors contributing to thrombocytopenia in ITP patients

**Figure 4:**
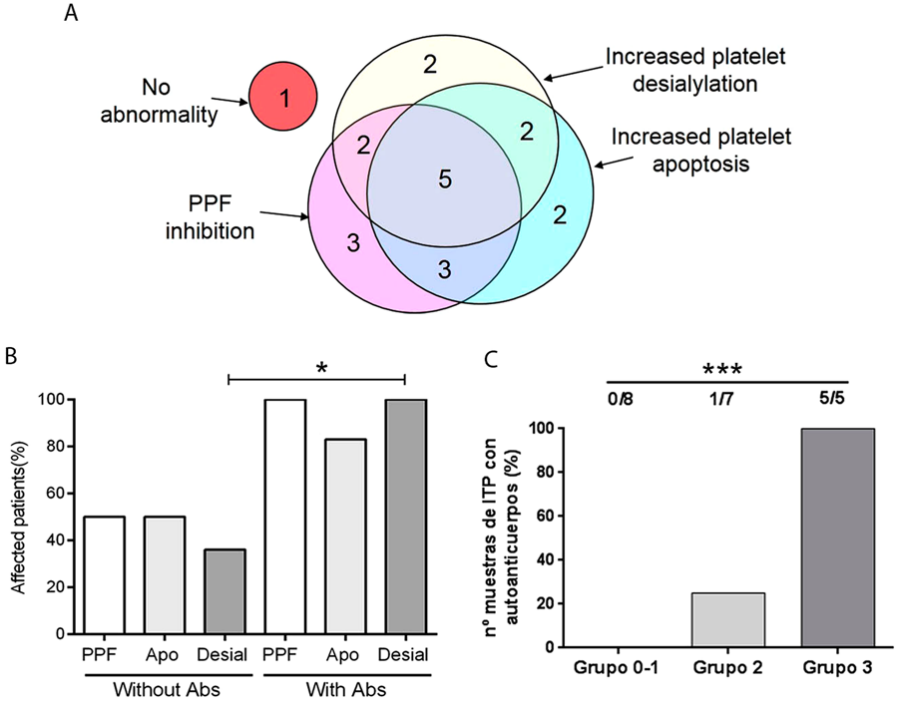
Frequency of abnormalities in ITP patients. (**A**) Venn diagram showing the number of patients/samples displaying increased platelet apoptosis, increased platelet desialylation and/or inhibition of proplatelet formation (PPF) in the ITP population studied (n = 20). (**B**) Incidence of platelet apoptosis (Apo), platelet desialylation (Desial) and inhibition of PPF (PPF) in patients according to the presence (with Abs) or absence (without Abs) of autoantibodies (Fisher’s exact test, *P < 0.05). (**C**) Percentage of ITP patients with detectable levels of platelet autoantibodies according to the number of defects observed (Group 0-1, patients harbouring none or only one defect, including inhibition in PPF, increased platelet desialylation or apoptosis; Group 2, patients with 2 abnormalities; Group 3, patients displaying impaired PPF, increased platelet desialylation and platelet apoptosis) (Chi-square test, ***P < 0.001).

Analysis of the contribution of thrombocytopenic factors to the pathophysiology of individual ITP patients revealed that 95% of the patients/samples had abnormalities in at least one of the parameters evaluated. However, distribution of these abnormalities did not follow a specific pattern, as some patients had only one defect, while others presented a combination of two or three of these alterations (Fig. 4A).

Concerning clinical and laboratory features, ITP patients with antiplatelet autoantibodies exhibited higher frequency of platelet desialylation (as evaluated by RCA binding) (Fisher’s exact test, P < 0.05). While inhibition of PPF and increased platelet apoptosis were more frequently observed in samples from patients bearing autoantibodies, differences between both groups were not statistically significant (Fig. 4B). In addition, patients displaying three abnormalities (group 3: inhibition of PPF, platelet apoptosis and platelet desialylation) exhibited higher frequency of autoantibodies than patients with two abnormalities (group 2) and those with one or none (group 0-1) of these features, (Chi-square test, P < 0.001) (Fig. 4C). Other variables such as platelet count, percentage of reticulated platelets, bleeding score, and splenectomy were similar in all groups (Table 1).

**Table 1:** Biochemical and clinical data of ITP patients.

Number of abnormalities	Group 0-1	Group 2	Group 3
Number of patients	8	7	5
Platelet count (10^9^/L)*	37,5 (22–65)	29.0 (8–62)	40.0 (20–53)
Average bleeding score	1,5	1,43	1,6
Percentage of patients with bleeding score** = 2	62%	57%	60%
Frequency of autoantibodies	0%	14%	100%
Reticulated platelets. (%)*	15.8 (8.6-32.3)	9.5 (3.0-20.0)	13.1 (5.5-18.7)
Treatment	62%	43%	100%
Splenectomy	12%	43%	40%

*Median and range are shown. **Bleeding score was evaluated according to the ITP Bleeding Scale (IBLS) proposed by Page and col. comprising 3 grades: from 0 (none) to 2 (marked bleeding) assessed at nine anatomical sites by history over the previous week^25^.

Regarding the possible influence of treatment on the evaluated effects, neither platelet apoptosis nor desialylation were different in the presence of samples from ITP patients undergoing treatment or not. Similar results were obtained for PPF. Beyond individual variable analysis, all patients who showed defects in the three parameters evaluated required treatment (Table 1). Interestingly, megakaryopoiesis was higher in the presence of plasma from ITP patients without treatment (n = 7) compared to that observed in the presence of samples from patients under treatment (n = 13) (p < 0.05).

## Discussion

In the present study, we analyzed the possible influence of different contributing factors leading to thrombocytopenia in ITP patients. Our results show inhibition of thrombopoiesis, platelet apoptosis and platelet desialylation as possible mechanisms underlying ITP. Some of these abnormalities coexisted in 60% of the patient samples studied, although we could not identify a particular common factor that links these pathways.

Of note, ITP plasma did not inhibit megakaryopoiesis. On the contrary, cell size, complexity and ploidy were unaltered while two samples (10%) induced an increase in MK count and nine samples (45%) increased MK CD42b expression. These results are in agreement with the fact that MK number in the bone marrow of ITP patients is often normal or increased^13^. Moreover, our results are consistent with previous data reported by Yang and colleagues^14^ showing that ITP plasma enhanced the number of megakaryocytes. Although there is no clear explanation for these findings, an increase of megakaryopoietic and/or inflammatory cytokines, or a decrease in antiproliferative factors in ITP plasma might represent potential mechanisms that contribute to increase MK number^15–17^. On the contrary, previous studies demonstrated inhibition of megakaryopoiesis in the presence of ITP plasma samples. Of note, there are clear methodological differences between these and our studies. While McMillan^7^ worked with 30-35% CD34+ cell purity and Chang^8^ used mononuclear cells from cord blood, in our working conditions, samples with at least 95% CD34+ cell purity were used. This methodology enables assessment of the direct effect of patient’s samples in megakaryopoiesis. The incorporation of a controlled number of specific bone marrow stromal and immune cells to the culture system could offer additional complex information. Remarkably, 78% of ITP samples that induced an increase in MK CD42b expression, displayed an inhibitory effect in the key step of proplatelet formation. Since our previous studies demonstrated that autoantibodies are mainly responsible for the inhibition of proplatelet formation, a possible explanation for the differential action of ITP samples on thrombopoiesis over megakaryopoiesis could arise from the fact that specific MK glycoproteins (i.e. GPIIbIIIa and GPIbIX) which are the main target of autoantibodies, play specific and central roles in thrombopoiesis^18,19^, different from those exerted during megakaryopoiesis, when these antigens are expressed. In addition, these findings reinforce the notion that autoantibodies specifically act at the step of thrombopoiesis and indicate that inhibition of platelet production in this setting is not related to inhibition of MK maturation.

Our results show RCA-I, a lectin that recognizes galactose residues, changes significantly in the presence of ITP plasma, in contrast to ECL and PNA which specifically bind to Galβ4GlcNAc and Galβ3GalNAc, respectively, and were less affected. Although RCA and ECL share some similarities in their glycan-binding specificities, significant differences have been described in both, affinity strength and detailed specificity^20^ that could account for the preferential binding of RCA to platelets exposed to ITP plasma. Moreover, changes in SNA, but not MAL II reactivity demonstrated that sialic acid linked to α2,6 position was more frequently lost than α2,3-linked sialic acid. A possible explanation for increased platelet desialylation could be an increase in platelet activation leading to neuraminidase externalization. However, platelet activation, as assessed by PAC-1 binding and P-selectin externalization was normal in our ITP population^3^, suggesting that loss of sialic acid may not be directly related to platelet activation, at least in our cohort. Previous studies by Li and colleagues^2^ showed that incubation of control platelets with anti-GPIbα ITP plasma triggered glycoprotein desialylation, while the effect of anti-GPIIbIIIa ITP plasma was moderate. In our ITP cohort, desialylation induced by ITP samples bearing anti-GPIIbIIIa autoantibodies was even higher than that observed in the presence of the anti-GPIb positive ITP sample, suggesting that platelet clearance by hepatocytes through Aswell-Morell receptors could take place in a wider population of ITP patients than previously reported. The precise glycoprotein receptor that is preferentially involved during this process still remains to be studied, although previous evidence suggested GPIb as a possible candidate given its heavy glycosylated composition^21^. Further studies using more sophisticated glycoanalytical technologies will be essential to fully dissect this mechanism.

As desialylation was linked to apoptosis in platelets incubated with anti-GPIb monoclonal antibodies, we speculated that a possible association might occur between these two abnormalities in our ITP group. As shown in Fig. 2I, 7 ITP samples showed both abnormalities. However, 4 samples induced desialylation while apoptotic parameters were unaffected in platelets and another 5 presented only apoptosis, suggesting that, at least in some patients, pathways leading to these abnormalities are completely independent.

One patient from our cohort did not show abnormalities in any of the cellular or biochemical pathways studied. However, this patient had similar platelet counts than patients displaying one, two or three abnormalities. Other factors leading to thrombocytopenia that were not evaluated in the present study such as a direct T-cell cytotoxic activity on platelets, complement-mediated lysis or antibody-mediated phagocytosis, might contribute to the low platelet count observed in this ITP patient.

Notably, the group of patients who had detectable autoantibody titters showed also higher incidence of alterations in every pathway studied, suggesting that autoantibodies might be the main cause of disturbances, both in platelet production and clearance.

MK apoptosis has been widely studied in ITP. However, results are not conclusive and opposing conclusions were reached. Houwerzijl and collaborators^22,23^ found ultrastructural abnormalities compatible with para-apoptosis in bone marrow MKs, while Yang and col^14^. described lower expression of tumour necrosis factor-related apoptosis-inducing ligand (TRIAL) and higher expression of anti-apoptotic BCL2L1 (Bcl-xL) in normal MKs incubated with ITP plasma. In the present study, neither ITP plasma alone nor ITP plasma together with normal immune cells (MNC) induced MK apoptosis, suggesting that this could not be a relevant mechanism that prevents platelet production in ITP.

Interestingly, the relevance of desialylation in MK physiology is unknown. Our current results show higher frequency of RCA binding to platelets (55%) as compared to MKs (5%) incubated with the same ITP samples, suggesting that desiaslylation may be a relevant mechanism of platelet clearance in ITP, although it does not affect MK fate. Further studies should be aimed at exploring whether changes in glycosylation may influence lectin binding and signaling in platelets of ITP patients^24^.

In our ITP cohort, 65% of patients were under treatment (92% under corticosteroid therapy). Although platelet apoptosis and desialylation as well as PPF results were similar in treated and untreated patients, megakaryopoiesis was higher in the presence of samples from non-treated patients. The analysis of a larger ITP cohort will help to determine whether this difference is actually related to treatment or if any other variable could influence megakaryopoiesis.

In conclusion, in this study we report different mechanisms that may account for low platelet count in the same group of ITP patients. Our results support the notion that causes of thrombocytopenia are multifactorial, and frequently involve concomitant defects in platelet production and clearance. These findings support the use of combination therapies, including drugs targeting different mechanisms of action, in patients with highly refractory disease.

## Materials and Methods

### Patients and blood samples

Twenty patients with chronic ITP (median age, 51 years, range 21–80) diagnosed according to current criteria^1^ were included. The study was approved by the Ethics Committee of Institute of Medical Research Alfredo Lanari. Clinical and laboratory data are presented in Table 2.

**Table 2:** Clinical and laboratory data from ITP patients.

Patient N°	Platelet count	Type of autoantibody			Bleeding	Treatment	Splenectomy
	(x10^9^/L)	Anti-GPIIbIIIa Anti-GPIbIX Anti-GPIaIIa			Score		
1	46				2	no	Yes
2	63				2	cortic	No
3	22				2	cortic	Yes
4	62				2	cortic	No
5	20				0	no	No
6	40	x			2	azatioprina	Yes
7	34	x			1	no	No
8	20		x		2	cortic	Yes
9	22				2	cortic	No
10	24	x			2	cortic	No
11	29				2	cortic	Yes
12	37				2	cortic	No
13	23				1	no	No
14	30				1	no	No
15	8				2	no	Yes
16	65				0	cortic	No
17	38				2	no	No
18	30				1	cortic	No
19	50	x			1	cortic	No
20	53	x		x	1	cortic	No

Blood samples were obtained as follows: 5 ml were collected into 342 mmol/L EDTA for cell count, 10 ml into 129 mmol/L sodium citrate to obtain recalcified plasma, and 5 mL into ACD (citric acid 71.4 mmol/L, sodium citrate 85 mmol/L, dextrose 11.1 mmol/L) for platelet isolation. Human cord blood was obtained following normal pregnancies and deliveries at Hospital Materno-Infantil Dr. Gianantonio. Product of leukapheresis were obtained from normal controls undergoing stem cell mobilization for allogeneic transplantation at Center of Medical Education and Clinical Research “Norberto Quirno” (CEMIC), Buenos Aires, Argentina. All samples were obtained upon written informed consent of donors, in accordance with the Declaration of Helsinki. Preparation of recalcified plasma and detection of autoantibodies using PAKAUTO kit (GTI Diagnostics Inc., Waukesha, WI, USA) were carried out as previously described^9^.

### Cell culture

CD34-positive cells were obtained from umbilical cord blood or from product of leukapheresis by immunomagnetic separation (Miltenyi Biotech Ltd., Bisley, Surrey, UK) as described previously^24^. Cultures with at least 95% CD34+ cells, as established by fluorescein isothiocyanate (FITC)-conjugated anti-CD34 binding (BD Bioscience, San José, CA, USA) were used.

### Evaluation of megakaryopoiesis in the presence of ITP plasma

CD34+ cells (2 × 10^4^) were cultured in StemSpan (Stem Cell Technologies, Vancouver, BC, Canada) with the addition of 10% recalcified patient or control plasma, 10 ng/ml Thrombopoietin and 10 ng/ml Interleukin 6 (both from Miltenyi Biotec Ltd). After incubation at 37 °C in a humidified atmosphere of 5% CO_2_ for 12 days, cells were counted and analysed by flow cytometry using FITC-conjugated anti-human CD61 and phycoerythrin (PE)-conjugated anti-human CD42b (BD Biosciences) to assess megakaryocyte differentiation and maturation. In order to evaluate cell size and complexity, mean forward scatter (FSC) and side scatter (SSC) were recorded. MK ploidy was assessed by flow cytometry, after 12-days culture of CD34+ cells with ITP or control plasma, by incubating 4 × 10^5^ cells with 50 µg/ml propidium iodide (Sigma-Aldrich) and 100 µg/ml RNase A (Sigma-Aldrich) overnight, using FITC-CD61 to identify the MK population.

### Evaluation of thrombopoiesis in the presence of ITP plasma

Thrombopoiesis in the presence of ITP plasma was assessed as described previously^9^.

### Platelet apoptosis

Evaluation of platelet apoptosis in ITP patients was carried out by measuring PS exposure, disruption of mitochondrial membrane potential, and active caspase 3 as previously described by Goette *et al*.^3^. Platelet apoptosis was defined as ‘abnormal’ when at least two of the three markers were above the normal limit reference range.

### PS exposure in normal MK incubated with ITP plasma

Normal mature MK obtained from umbilical cord blood derived-CD34+ cells at day 13 of culture were incubated with 10% recalcified ITP or normal plasma for 24 h. Then, samples were labelled with FITC-annexin V and acquired within 1 h on a FACSCanto II flow cytometer (BD Biosciences). MKs were identified by their CD41 positivity. Studies were carried out using MKs from at least two cord blood samples. To test whether ITP plasma could induce PS exposure in normal MKs through an indirect effect exerted by normal autologous immune cells, CD34+ hematopoietic progenitors obtained from peripheral blood of mobilized normal subjects were cultured during 12 days to obtain mature MKs as described. Then, purified mononuclear cells (MNC) from the same source were added to the culture media in a 1:2 proportion (MK:MNC) and the culture was supplemented with 10% ITP or control plasma. After 24 h incubation, FITC-Annexin V binding was assessed by flow cytometry within the CD41+ population.

### Assessment of desialylation in normal platelets and MK incubated with ITP plasma

To evaluate changes in platelet sialylation induced by ITP plasma samples, PGE_1_ was added to normal platelet rich-plasma and platelets were subsequently washed. Platelets (2.5 × 10^5^) were resuspended in 50 µl of either ITP or control plasma and incubated for 1 h at 22 °C. To evaluate MK desialylation, two strategies were used: 1) mature MK were obtained from normal CD34+ plasma-free cell culture with the addition of 10 ng/ml thrombopoietin (Tpo) and interleukin 6 (IL-6) for 12 days. Then, 10% ITP or control plasma were added to the culture media and further incubated for 1 hour; 2) normal CD34+ cells (day 1 of culture) were incubated for 12 days in the presence of 10 ng/ml Tpo and IL-6, with the addition of 10% ITP or normal recalcified plasma. After washing, cells (either platelets or MK) were incubated with 5 μg/ml FITC-RCA-I that binds to galactose residues present in membrane glycoconjugates (Vector Laboratories, Burlingame, CA, USA) for 20 min. Additionally, normal platelets incubated with ITP or control plasma were incubated with 16.7 μg/ml ECL, or 20.0 μg/ml PNA, to evaluate cell surface Galβ4GlcNAc and Galβ3GalNAc, respectively. α2,6- or α2,3-linked sialic acid was detected using 6.7 μg/ml SNA and 5.0 μg/ml MAL II, respectively (biotinylated ECL, PNA, MALII and SNA lectins were all from Vector Laboratories). After a washing step, a final incubation with streptavidin-allophycocyanin (APC) (BioLegend, San Diego, CA, USA) was carried out. Cells were acquired on a FACSAria flow cytometer (BD Biosciences) as previously described. PE-labelled anti-human CD41 or FITC-labelled anti-human CD42a monoclonal antibodies (BD Biosciences) were used to identify platelet and megakaryocytic populations and either FITC or APC mean fluorescence intensity (MFI) was recorded for each sample. Samples were tested at least twice using different normal platelet donors or cord blood-derived MK.

### Reticulated platelets

Reticulated platelets were determined by flow cytometry using thiazole orange (TO, Sigma-Aldrich, St Louis, MO, USA). Briefly, platelets were incubated with TO, 10 ng/mL for 1 hour at 22 °C in the dark. Platelets were identified using PE-conjugated anti-CD41.

### Statistical analysis

Data are presented as mean ± SD or median and range. Reference values were established as the mean ± 2 SD of control samples. Variables were analysed for normality and equality of variances using Shapiro-Wilks and F-test, respectively. Differences between data from ITP samples and normal controls was assessed using unpaired *t*-test or Mann–Whitney test. Differences between data from three groups were assessed by one-way ANOVA followed by Tukey’s multiple comparisons test. Frequency distribution among two or three groups were studied by Fisher’s exact test and Chi square test, respectively. P values less than 0.05 were considered statistically significant.

## Supplementary information

Supplementary information

## Data Availability

Data generated and/or analysed during the current study are available from the corresponding author on reasonable request.
