# Publisher Correction: Multiple concomitant mechanisms contribute to low platelet count in patients with immune thrombocytopenia

**DOI:** 10.1038/s41598-020-58553-y

**Published:** 2020-02-12

**Authors:** Matías Grodzielski, Nora P. Goette, Ana C. Glembotsky, M. Constanza Baroni Pietto, Santiago P. Méndez-Huergo, Marta S. Pierdominici, Verónica S. Montero, Gabriel A. Rabinovich, Felisa C. Molinas, Paula G. Heller, Paola R. Lev, Rosana F. Marta

**Affiliations:** 1 University of Buenos Aires, Institute of Medical Research A Lanari, Buenos Aires, Argentina; 2 Department of Experimental Hematology, Institute of Medical Research (IDIM), National Scientific and Technical Research Council (CONICET), University of Buenos Aires, Buenos Aires, Argentina; 3 Laboratory of Immunopathology, Institute of Biology and Experimental Medicine (IBYME), National Scientific Council of Scientific and Technical Research (CONICET), C1428 Buenos Aires, Argentina; 4 Department of Hematology, Ramos Mejía Hospital, Buenos Aires, Argentina; 5 Department of Biochemistry, Center of Medical Education and Clinical Research “Norberto Quirno” (CEMIC), Buenos Aires, Argentina; 6 Department of Biological Chemistry, School of Exact and Natural Science, University of Buenos Aires, C1428 Buenos Aires, Argentina

Correction to: *Scientific Reports* 10.1038/s41598-018-38086-1, published online 18 February 2019

The original version of this Article contained extensive errors in the Reference list. References 13–23 were incorrectly listed as references 14–24 respectively, and reference 24 was incorrectly listed as reference 13.

In addition, reference 26 was omitted and is listed below:

Page L.K. *et al*. The immune thrombocytopenic purpura (ITP) bleeding score: assessment of bleeding in patients with ITP. *Br J Haematol*, **138**, 245–8 (2007).

Finally, the original version of this Article contained errors in the in-text citations.

In the legend for Table 1,

“*Median and range are shown. **Bleeding score was evaluated according to the ITP Bleeding Scale (IBLS) proposed by Page and col. comprising 3 grades: from 0 (none) to 2 (marked bleeding) assessed at nine anatomical sites by history over the previous week^25^.”

now reads:

“*Median and range are shown. **Bleeding score was evaluated according to the ITP Bleeding Scale (IBLS) proposed by Page and col. comprising 3 grades: from 0 (none) to 2 (marked bleeding) assessed at nine anatomical sites by history over the previous week^26^.”

In the Materials and Methods, under the subheading ‘Cell culture’,

“CD34-positive cells were obtained from umbilical cord blood or from product of leukapheresis by immunomagnetic separation (Miltenyi Biotech Ltd., Bisley, Surrey, UK) as described previously^24^.”

now reads:

“CD34-positive cells were obtained from umbilical cord blood or from product of leukapheresis by immunomagnetic separation (Miltenyi Biotech Ltd., Bisley, Surrey, UK) as described previously^25^.”

These errors have now been corrected in the HTML and PDF versions of the Article.

