# Supplementary material for: Multiple concomitant mechanisms contribute to low platelet count in patients with immune thrombocytopenia
Source: Sci Rep. 2019 Feb 18;9:2208. doi: 10.1038/s41598-018-38086-1 (PMC6379541; doi:10.1038/s41598-018-38086-1)
Supplement: Supplementary file 1 — Supplementary information [file 41598_2018_38086_MOESM1_ESM.docx]

**Multiple concomitant mechanisms contribute to low platelet count in patients with immune thrombocytopenia**

Matías Grodzielski^1,2^, Nora P Goette^1^, Ana C Glembotsky^1,2^, M. Constanza Baroni Pietto^1,2^, Santiago P Méndez-Huergo^3^, Marta S Pierdominici^4^, Verónica S Montero^5^, Gabriel A Rabinovich^3,6^, Felisa C Molinas^1,2^, Paula G Heller^1,2^, Paola R Lev^1,2 *^and Rosana F Marta^1,2*^.

Supplementary Results

Studies using CD34+ cells from cord blood were reproduced using CD34+ cells from peripheral blood mononuclear cells from normal subjects who were stimulated with growth factors for allogeneic bone marrow transplantation. (Supplementary Table S1). Similarly to results obtained using CD34+ cells from umbilical cord blood, ITP plasma did not inhibit megakaryopoiesis in these conditions.

Supplementary Table S1

Megakaryocyte differentiation and maturation from CD34+ hematopoietic progenitors obtained from peripheral blood of mobilized normal subjects.

|  | ITP patients | Controls |
| --- | --- | --- |
| Total MK count | 57772 ± 50050 | 18428 ± 15403 |
| Mature MK count | 43884 ± 39979 | 12202 ± 11131 |
| CD61 MFI | 158,9 ± 40,82 | 123,8 ± 20,99 |
| CD42b MFI | 145,1 ± 31,99 | 124,8 ± 19,40 |
